# Protein-protein interactions of the hyperthermophilic archaeon *Pyrococcus horikoshii *OT3

**DOI:** 10.1186/gb-2005-6-12-r98

**Published:** 2005-11-18

**Authors:** Kengo Usui, Shintaro Katayama, Mutsumi Kanamori-Katayama, Chihiro Ogawa, Chikatoshi Kai, Makiko Okada, Jun Kawai, Takahiro Arakawa, Piero Carninci, Masayoshi Itoh, Koji Takio, Masashi Miyano, Satoru Kidoaki, Takehisa Matsuda, Yoshihide Hayashizaki, Harukazu Suzuki

**Affiliations:** 1 CREST, Japan Science and Technology Agency, 4-1-8 Honcho, Kawaguchi, Saitama 332-0012, Japan; 2 Laboratory for Genome Exploration Research Group, RIKEN Genomic Sciences Center (GSC), 1-7-22 Suehiro-cho, Tsurumi-ku, Yokohama 230-0045, Japan; 3 Genome Science Laboratory, RIKEN, 2-1 Hirosawa, Wako 351-0198, Japan; 4 Highthroughput Factory, RIKEN Harima Institute, 1-1-1 Kouto, Mikazuki-cho, Sayo-gun, Hyogo 679-5148, Japan; 5 Structural Biophysics Laboratory, RIKEN Harima Institute, 1-1-1 Kouto, Mikazuki-cho, Sayo-gun, Hyogo 679-5148, Japan; 6 Division of Biomedical Engineering, Graduate School of Medicine, Kyushu University, Fukuoka 815-8582, Japan

## Abstract

**Background:**

Although 2,061 proteins of *Pyrococcus horikoshii *OT3, a hyperthermophilic archaeon, have been predicted from the recently completed genome sequence, the majority of proteins show no similarity to those from other organisms and are thus hypothetical proteins of unknown function. Because most proteins operate as parts of complexes to regulate biological processes, we systematically analyzed protein-protein interactions in *Pyrococcus *using the mammalian two-hybrid system to determine the function of the hypothetical proteins.

**Results:**

We examined 960 soluble proteins from *Pyrococcus *and selected 107 interactions based on luciferase reporter activity, which was then evaluated using a computational approach to assess the reliability of the interactions. We also analyzed the expression of the assay samples by western blot, and a few interactions by *in vitro *pull-down assays. We identified 11 hetero-interactions that we considered to be located at the same operon, as observed in *Helicobacter pylori*. We annotated and classified proteins in the selected interactions according to their orthologous proteins. Many enzyme proteins showed self-interactions, similar to those seen in other organisms.

**Conclusion:**

We found 13 unannotated proteins that interacted with annotated proteins; this information is useful for predicting the functions of the hypothetical *Pyrococcus *proteins from the annotations of their interacting partners. Among the heterogeneous interactions, proteins were more likely to interact with proteins within the same ortholog class than with proteins of different classes. The analysis described here can provide global insights into the biological features of the protein-protein interactions in *P. horikoshii*.

## Abstract 2

Protein-protein interactions among 960 *Pyrococcus *soluble proteins have been analysed by mammalian two-hybrid analysis and thirteen interactions between annotated and unannotated proteins detected.

## Background

*Pyrococcus horikoshii *OT3, a hyperthermophilic anaerobic archaeon, isolated in 1992 from a hydrothermal vent at a depth of 1,395 m in the Okinawa Trough in the Pacific Ocean, grows at temperatures ranging from 85°C to 100°C, and optimally at 98°C [1]. The complete genome sequence of *P. horikoshii *OT3 has been determined: a total of 2,061 open reading frames (ORFs) were assigned over the entire genome sequence of 1,708,505 base pairs [2]. According to Kawarabayasi *et al*. [2], a sequence homology search showed that 557 (27.0%) of the ORFs exhibited similarity to characterized genes in other organisms, and that more than half, 1,049 ORFs (50.9%), showed no significant similarity to any sequence in public databases: 455 (22.1%) showed significant similarity only to uncharacterized proteins. As assignment of ORFs is just a prediction, whether actual protein expression occurs from each uncharacterized ORF has yet to be confirmed. Thus, to better understand the mechanisms that allow this organism to live in such an extreme environment, it is necessary to analyze the functions of the uncharacterized proteins.

In uncovering the functions of proteins, systematic examination of protein-protein interactions (PPIs) is important. Because most proteins operate as parts of complexes to regulate biological processes in cells or entire organisms, PPIs enable us to predict the functions of uncharacterized proteins through their associations with proteins of known function [3,4]. Although many approaches are used to examine PPIs, two-hybrid systems have been applied to a wide variety of organisms, such as viruses [5-7], eukaryotes [8-13], and eubacteria [14-16]; however, no large-scale archaeal PPI analysis by any method has yet been reported. A particularly interesting question is whether the PPIs in the hyperthermophilic *P. horikoshii *OT3 are similar to other organisms, or unique. Here, we used our mammalian two-hybrid system [11] to conduct a large-scale PPI analysis of the intracellular and soluble proteins of *P. horikoshii *OT3.

## Results

### Protein-protein interaction analysis

**Figure 1:**
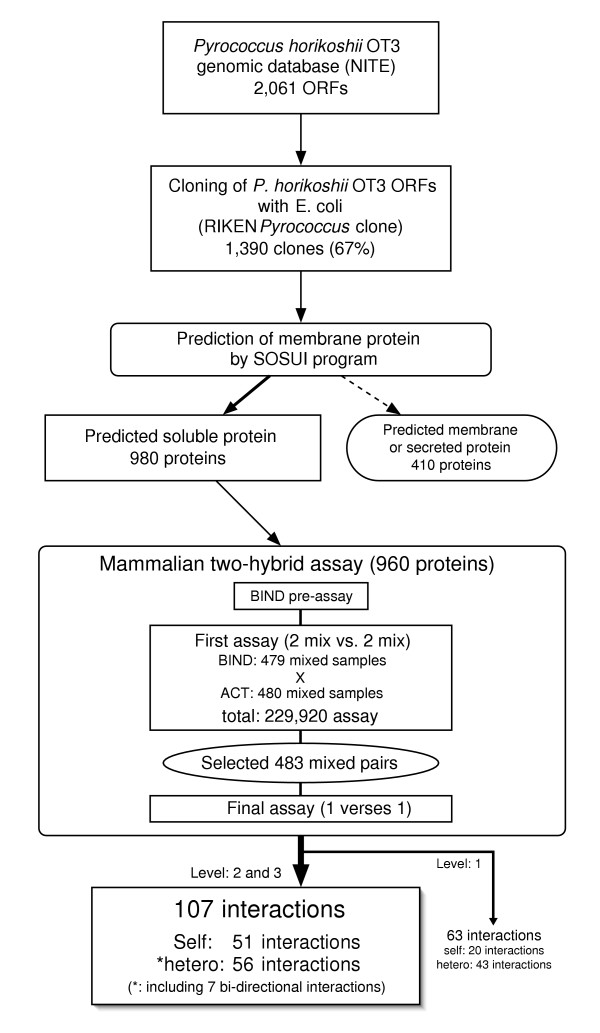
Flow chart of the screening process used to identify *P. horikoshii *OT3 protein-protein interactions. In this flow chart the construction of BIND and ACT samples by PCR is omitted. We selected 980 predicted soluble proteins using the SOSUI program [17], and applied 960 of 980 clones for the first assay. The details are given in Materials and methods and Additional data file 1. ACT, VP16 transcriptional activation domain; Bind, Gal4 DNA-binding domain; ORF, open reading frame.

The PPIs of *P. horikoshii *OT3 were explored using the mammalian two-hybrid system that we had already established [11]. A flow chart of the assay process is shown in Figure 1. *P. horikoshii *OT3 has 2,061 ORFs and we cloned 1,390 of these (data not shown). These clones were the starting material for our analysis. The protein interactions of membrane proteins and secreted proteins are generally hard to analyze using the two-hybrid system because the process occurs in the nucleus. Using the SOSUI program [17], we examined the ORFs to deduce which proteins included membrane-spanning sequences or signal peptide sequences; 410 clones were removed because they were predicted to code for membrane or secreted proteins.

The basis of the mammalian two-hybrid assay is the bait and prey protein interaction, where interaction initiates transcription of the luciferase reporter gene; an increase in expression of the luciferase reporter gene corresponds to the interaction between the bait and prey proteins. Assay samples expressing bait and prey proteins (Gal4- and VP16-fusion proteins, respectively) were constructed by PCR (see Materials and methods, and Additional data file 1). We pooled the samples for the first assay. As two bait samples were excluded from the assay due to self-activation, a total of 479 two-mixture (two-mix) bait samples and 480 two-mix prey samples were systematically tested in the first assay. The positive combinations in the first assay were then examined using combinations of single bait and prey samples to identify the interacting pairs. We finally obtained 170 interactions from the assay.

### Assessment of the protein-protein interaction analysis

**Figure 2:**
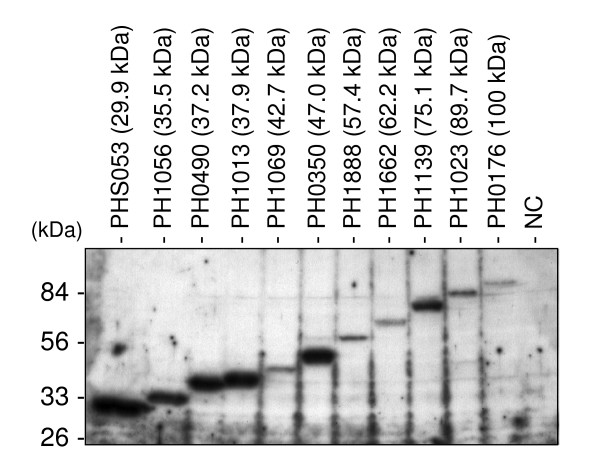
Expression of the assay samples. The *P. horikoshii *fusion proteins, expressed in CHO-K1 cells from 34 randomly selected bait samples, were evaluated by western blot analysis using a polyclonal antibody against the Gal4 DNA binding domain. In this figure, we show the representative results of 11 samples. The cell lysate without transfection was used as a negative control (NC). Each molecular size with the gene ID (PHXXXX) corresponds to the Gal4-fusion protein.

The frequency of self-activation proteins in *P. horikoshii *OT3 was 0.2% in our mammalian two-hybrid system, which is relatively lower than the frequencies of self-activation proteins observed in other organisms [9-12,16]. One of the reasons for this might be that many of the *Pyrococcus *proteins were not sufficiently expressed in our system because of different preferences for codon usage between *P. horikoshii *and mammalian cells. Thus, we randomly selected 34 bait samples and examined expression of the fusion proteins in CHO-K1 cells using western blot analysis. All of the samples exhibited signals with expected molecular size (Figure 2; the data shows the representative results of 11 samples). However, the amount of detected proteins seems to be dependent on molecular size; proteins with a size less than about 50 kDa showed strong signals whereas there was a tendency for larger proteins to show relatively weaker signals.

**Figure 3:**
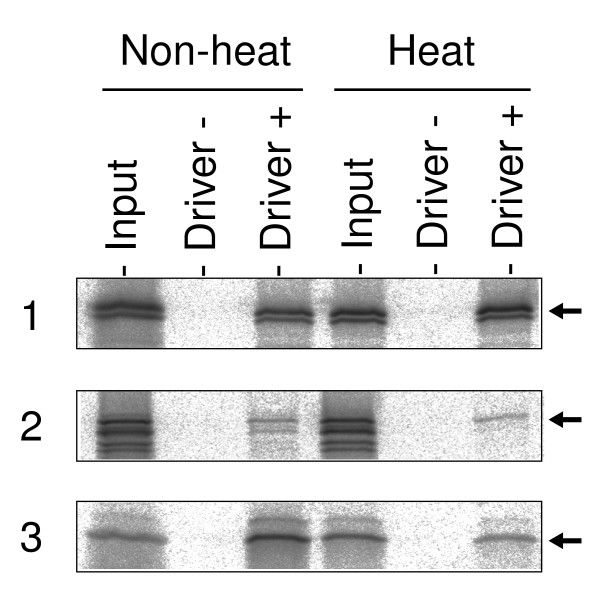
Observation of *Pyrococcus *protein-protein interactions *in vitro *and the effect of heat pre-incubation on synthesized proteins. An *in vitro *pull-down assay was carried out after incubation of proteins at 37°C (non-heat) or 75°C (heat). The results with or without biotinylated driver proteins are shown as 'driver +' or 'driver -', respectively, and 10% of the ^35^S-labeled proteins in the assay were treated as 'input'. The three hetero-interaction pairs that were biotinylated and ^35^S-labeled are as follows: lane 1, PH1354 and PH1355; lane 2, PH0795 and PH0017; lane 3, PH0245 and PH1553. Arrows indicate bands of ^35^S-labeled proteins that were precipitated with biotinylated interacting partners.

We detected characteristic hetero-interactions consisting of α and β subunit proteins, such as indolepyruvate ferredoxin oxidoreductase (PH1138-PH0229 or PH1138-PH0764), and 20S proteasome (PH1553-PH0245), showing that we successfully identified at least some of the protein interactions in *P. horikoshii *OT3. Several reports indicate, however, that recombinant *Pyrococcus *proteins expressed in *Escherichia coli *cells assume their mature conformations or activities only after heat activation [18,19], suggesting that the other interactions might be artificial and are only detected at 37°C, the temperature used in our analysis method. Thus, we evaluated the interactions derived from our mammalian two-hybrid system using the *in vitro *pull-down assay, with or without heat activation. In three of the protein pairs, all of the ^35^S-labeled proteins were successfully precipitated (Figure 3), showing that these protein pairs can interact with each other regardless of heat activation. The results indicate that at least some *Pyrococcus *proteins form their native conformations when expressed in cultured mammalian cells at 37°C.

### Selection of reliable interactions

Generally, PPIs obtained from the two-hybrid method have many false positives, which may complicate elucidation of the biological importance of the interactions. For further analysis it is best to select reliable interactions using positive and negative training interaction sets that can be made from known interaction information, which was done in the analysis of *Drosophila *interactions [9]. This approach seems impossible to apply to the *Pyrococcus *interactions, however, because few of the PPIs in *P. horikoshii *OT3 or related species are known. To overcome this problem, we considered the activity of the luciferase reporter gene. In our mammalian two-hybrid system, the activity of the luciferase reporter is used to judge protein interactions. We classified the interactions into levels 1 to 3 (weak to strong) depending on the strength of luciferase activity (see Materials and methods). We decided to take the level 2 and 3 interactions as the selected interaction set, resulting in 107 interactions consisting of 51 self-interactions and 56 hetero-interactions, including 7 bi-directional interactions (Figure 1).

**Figure 4:**
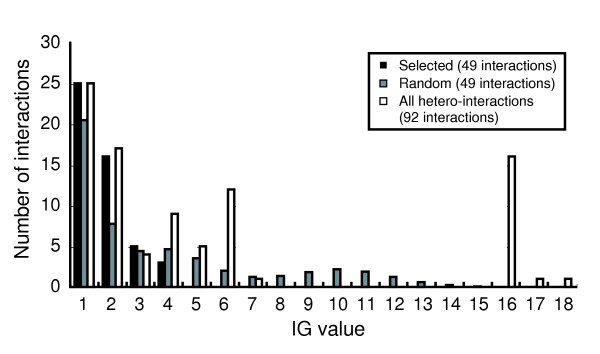
Selection and evaluation of the *P. horikoshii *OT3 protein-protein interactions. The distribution of interaction generality (IG) values, the reliability score, is shown for the 'all interaction' set (92 independent hetero-interactions) and the 'selected' interaction set (49 independent hetero-interactions), from which we removed interactions with a luciferase reporter activity level of 1. 'Random' shows the IG distribution of computationally calculated trials where we randomly removed the same number of interactions.

**Figure 5:**
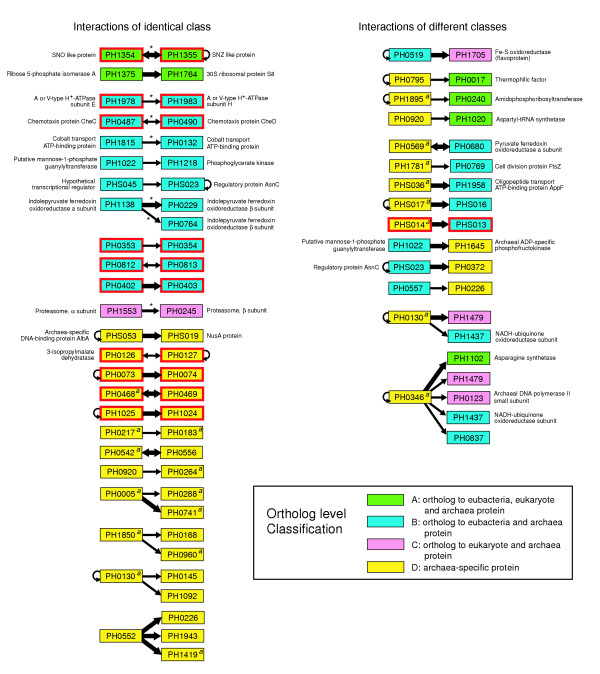
The selected hetero-interactions. The gene ID refers to that of the DOGAN genome database of *P. horikoshii *OT3 [26]. Ortholog classification was performed using the Sequence Similarity Database (SDDB) [46] of the Kyoto Encyclopedia of Genes and Genomes (KEGG) [48,49]. Proteins are classified and color-coded by ortholog level. The arrow in each interaction indicates the direction of bait protein to prey protein in the mammalian two-hybrid assay. The luciferase reporter activity of an interaction is indicated by thin lines (level 2) or thick lines (level 3). Annotations were derived using the SSDB [46] with entries for the *P. horikoshii *OT3 genome in KEGG [48,49] or the Database of Genomes Analyzed in NITE (DOGAN) [26]. Interactions marked with an asterisk indicate the existence of interlogs in other organisms. The protein pairs marked with red frames were encoded as operons on the *P. horikoshii *OT3 genome. '*a*' marks proteins unique to *Pyrococcus *without orthologs in other archaeal species.

We evaluated this selected interaction set using the interaction generality (IG) measure, a method for computationally assessing the reliability of PPIs [20]. Interactions with lower IG values are more likely to be reliable than interactions with higher IG values. The IG values for all interactions ranged from 1 to 18, whereas the IG values for the selected interaction set ranged from 1 to 4 (Figure 4). The average IG value of 5.52 for all interactions decreased significantly to 1.71 in the selected set. The latter value is also significantly lower than the average IG value of 3.80 (*P* < 0.0001) that was calculated from 10,000 mathematical trials that randomly removed the same number of interactions (by a jack-knife calculation). Furthermore, of the seven *P. horikoshii *OT3 hetero-interactions for which we found corresponding interactions in other species (interlogs), all were in the selected interaction set (asterisks in Table 1 and Figure 5). The result suggests that we successfully concentrated the true-positive interactions.

### Protein-protein interactions from the corresponding ORF pairs mapped to adjacent loci on the *P. horikoshii *OT3 genome

**Figure 6:**
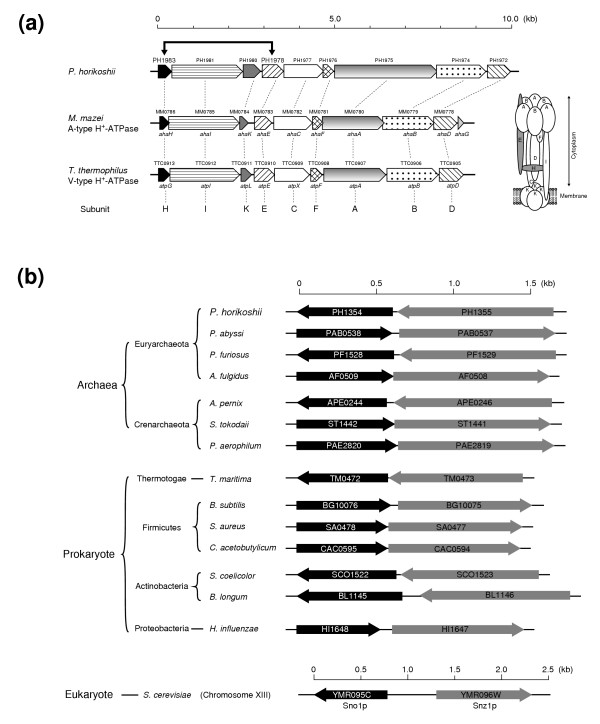
Alignment of two gene clusters related to the *P. horikoshii *OT3 protein-protein interactions. All the gene clusters were obtained using the Sequence Similarity Database (SDDB) [46] gene cluster search of the Kyoto Encyclopedia of Genes and Genomes (KEGG) [48,49]. **(a) **The gene cluster encoding archaeal- or vacuolar-type H^+^-ATP synthase protein subunits. The arrow shows the *P. horikoshii *protein interaction pair PH1983 and PH1978. The structure model (inset) was derived from *Methanococcus jannaschii *[22]. **(b) **The gene cluster encoding SNO/SNZ family proteins similar to PH1354 and PH1355. The black and gray open reading frames correspond to SNO and SNZ proteins, respectively.

In *P. horikoshii *OT3, the IDs of the predicted ORFs are systematically numbered according to their genomic location. We mapped the ORFs of the interacting protein pairs onto the *P. horikoshii *OT3 genome and found that in 11 out of 49 hetero-interactions (22%), the corresponding ORF pairs were mapped to adjacent loci on the genome, and that several other ORFs located close were orientated in the same direction (Table 2; Figure 6). We hypothesize that these ORF pairs belong to operons, where many of the functionally related genes in eubacteria and archaea are transcribed as a polycistronic mRNA.

To confirm our hypothesis, we explored the interlogs of the *Pyrococcus *interactions (Figure 6). The ORFs corresponding to the interacting pair PH1978 and PH1983 are separated on the OT3 genome by the ORFs for PH1981 and PH1980; although close to one another, and close to five downstream ORFs (PH1972, PH1974, PH1975, PH1976, and PH1977), all the ORFs are encoded in the same direction. The proteins PH1983 to PH1972 all show high similarity to archaeal-type H^+^-ATP synthase protein subunits in *Methanosarcina mazei *and *Methanococcus jannaschii*, and vacuolar-type H^+^-ATP synthase protein subunits in *Thermus thermophilus *(Figure 6a). In *M. mazei*, the gene cluster for these proteins is reported to be organized into a single operon [21]. In addition, the structure of the protein complex of this ATP synthase has been reported in *M. jannaschii *[22], where subunits E and H, corresponding to PH1978 and PH1983, interact directly on the intracellular side (Figure 6a inset). It is highly plausible, therefore, that this ORF cluster in OT3 is in an operon. We also found that the interacting pair of PH0487 and PH0490 (not annotated) have high similarity to the *Bacillus subtilis *chemotaxis proteins CheC and CheD, the genes for which are located adjacently on the *Bacillus subtilis *genome and compose an operon [23], suggesting that the ORFs for PH0487 and PH0490 are also expressed in *P. horikoshii *OT3 as an operon and that their functions are similar to those of CheC and CheD.

Another example is PH1354 and PH1355, which are similar to SNO1 (32% identity) and SNZ1 (56% identity), respectively, from *Saccharomyces cerevisiae *(Figure 6b). In the Pfam database (The Sanger institute, UK) [24], SNO1 and SNZ1 are shown to be widely conserved proteins categorized as SNO (PF01174) and SNZ family (PF01680) proteins. Members of these families are related to the pyridoxine biosynthetic pathway and are ethylene-responsible proteins, or glutamine aminotransferases. All known orthologous *SNO*/*SNZ *genes in eubacteria and archaea are adjacently located in the same direction on the genome, suggesting that they are likely to be organized in an operon (Figure 6b). Further, it has been shown using the yeast two-hybrid method and DNA microarray analyses, that SNZ1 and SNO1 in *S. cerevisiae *interact with each other and that their genes are co-regulated [25].

### Annotation of the interacting proteins in *P. horikoshii *OT3

As very few proteins in *P. horikoshii *OT3 have had functions assigned to them, we predicted the functions of the interacting proteins by using the annotations from orthologous proteins. We classified all of the 960 explored *P. horikoshii *OT3 proteins into 4 classes depending on the existence of orthologous proteins: class A, proteins with eubacteria, eukaryote and archaea orthologs (177); class B, proteins with eubacteria and archaea orthologs (228); class C, proteins with eukaryote and archaea orthologs (69); and class D, archaea-specific proteins (486). The set of selected self-interacting proteins (Table 1) and the set of selected hetero-interacting proteins (Figure 5) were also arranged according to these classes and by the annotation information of orthologs. Gene IDs for the hetero-interacting proteins are available in the DOGAN database of *P. horikoshii *OT3 [26].

### Characteristics of the self-interacting proteins

Of the selected 51 self-interacting proteins, 20 of 29 proteins in classes A to C were well annotated, whereas for the archaea-specific proteins in class D, only 1 out of 22 proteins was annotated (Table 1). The proteins in classes A and B were often annotated as enzymes involved in cellular metabolism, which is consistent with previous reports that many of these enzymes form homo-oligomer structures [27-33]. PH0119 and PHS042 in class C are not enzymes; the first is similar to the DNA repair RadA/Rad51 protein, and the second to the RNA-binding small nucleoprotein (Sm protein), which are both known to form homo-heptamer structures [34,35]. PHS053 was the only annotated protein in class D, showing similarity to archaea-specific DNA-binding protein AlbA of *Archaeoglobus fulgidus*, which has been shown by X-ray crystallography to form a homo-dimer structure and to possess DNA-binding properties [36].

### Characteristics of the hetero-interacting proteins

As with the self-interacting proteins, many of the hetero-interacting proteins in classes A to C were well annotated, but few of the class D archaea-specific proteins were described (Figure 5). More than half of the hetero-interactions between proteins of the same class consisted of the archaea-specific protein pairs (17 out of 30 interactions), and in the interactions between proteins of different classes, most of the pairs included archaea-specific proteins as one of the interaction partners (18/19 interactions, 95%). In the interactions for which both proteins in a pair are annotated, the annotations are related. For instance, PH1022-PH1645 are orthologs of proteins related to sugar phosphate metabolism, mannose-1-phosphate guanylyltransferase and ADP-specific phosphofructokinase, respectively. These results are reasonable as many proteins play a role in the network of cellular biological processes by associating with other related proteins (guilt-by-association) [3]. Based on this concept, several research groups have successfully predicted the functions of uncharacterized proteins using data on their interaction with other proteins [10-13,37]. We obtained 13 hetero-interactions between annotated proteins and hypothetical proteins (Figure 5), in which the functions of such hypothetical proteins are likely to be related to the functions of their interaction partners.

Dividing the hetero-interactions into two groups according to the classes described above - hetero-interactions consisting of interactions between proteins of the same class (30 pairs, 61.2%) and interactions between proteins of different classes (19 pairs, 38.8%) (Figure 5) - we found that the percentage of hetero-interactions consisting of interactions between proteins of the same class was significantly (*P* < 0.01) higher than the expected value of 35.1%, which was calculated by assuming that the interactions are not biased by class.

## Discussion

In this study, we report the systematic analysis of PPIs in *P. horikoshii *OT3 using our mammalian two-hybrid system. This is the first systematic analysis of PPIs in this hyperthermophilic archaeon. We successfully identified 170 interactions from 960 samples. From these, we selected 107 interactions (including 7 bi-directional interactions) according to luciferase reporter activity and evaluated them using the IG method. Detecting the interaction of hyperthermophilic proteins at 37°C may be a major drawback in this large-scale examination, there being no alternative with the present-day technology for gene manipulation in hyperthermophiles. We showed using western blot analysis and *in vitro *pull-down assays, however, that most of the *Pyrococcus *proteins could be expressed sufficiently in cultured mammalian cells at 37°C, in which at least some of the proteins seem to form their native conformations. In addition, some of the obtained interactions have been observed in other organisms (marked with asterisks in Table 1 and Figure 5). Many of the self-interacting proteins were enzymes. This tendency also supports our results and has been observed in other species [27-33]. Together with these results, it is reasonable to expect that many of the obtained interactions reflect functional, *in vivo *interactions.

Interacting proteins are likely to be encoded in the same operon [38]; of 49 independent hetero-interactions, we identified 11 hetero-interactions belonging to the same operons. Similar results have also been reported for the PPIs of *Helicobacter pylori*, in which the genomic localization of genes in interacting pairs was used to predict the functions of uncharacterized proteins [16]. Interestingly, we also found that protein pairs encoded in the same operon (marked by red frames in Figure 5) were much more frequent in the hetero-interactions between proteins of the same class than in the hetero-interactions between proteins of different classes (10 to 0, respectively, *P* < 0.02). This result suggests that interacting proteins in the same operon are more likely to evolve at similar rates.

Classifying the *Pyrococcus *proteins according to their homology data enabled us to better annotate them and characterize their interactions. We obtained many protein interactions between the archaea-specific proteins and between the archaea-specific proteins and other classes of proteins. It will be interesting to analyze the structures of such archaea-specific interacting proteins because they may possess novel protein interaction domains. Alternatively, although we did not observe any known domains in these proteins from their primary amino acid sequences, such proteins may possess novel domains that are structurally quite similar to known ones, as suggested by other reports [39,40]. We also found that the number of hetero-interactions between proteins of the same class was significantly more than the expected value. This observation may be explained by postulating that the protein interactions essential for many organisms are preferentially conserved beyond three kingdoms. Such interacting proteins may evolve at similar rates and show slower evolutionary changes than other proteins because substitutions in one protein would result in selection pressure for reciprocal changes in the interacting partners. This postulation has been generally confirmed [41].

## Conclusions

We analyzed 960 soluble proteins of *P. horikoshii *OT3 using the mammalian two-hybrid system, and found 107 reliable PPIs. Furthermore, proteins in the identified interactions were classified by ortholog level, and we found a trend that proteins were more likely to interact with proteins within the same ortholog class than with proteins from different classes. Although we could not identify a large amount of protein interactions in our assay, the data are still valuable for several reasons.

We found thirteen unannotated proteins that interacted with previously annotated proteins. These interaction data are useful for predicting the functions of the unannotated proteins from the annotations of their interacting partners; a prediction that could not be achieved by the analysis of operons because most of the protein pairs (12 out of 13 interactions) are not in the same operon. This information is important because many proteins of *P. horikoshii *OT3 have no similarity to proteins from other organisms and have not been annotated yet [2]. We must be careful, however, in making predictions based on results from imperfect single two-hybrid interactions.

We were able to predict the location of several operons and the hetero-interactions we identified support these predictions. This is valuable for annotating other hypothetical proteins involved in the same operons as proteins encoded in the same operon are closely related to one another functionally [42].

The interactions between unannotated proteins suggest that the corresponding ORFs are expressed as functional proteins. Many of the currently predicted ORFs on the *P. horikoshii *genome have not been evaluated as to whether they express actual proteins [2].

The data will contribute to the project analyzing the structures of *P. horikoshii *OT3 proteins using NMR and X-ray crystallography that has recently started [43]. The interaction data provide information indicating that the interacting proteins may possess native structures without heat activation, even when expressed at 37°C. In addition, for structural analysis to be successful, some proteins may have to be treated as complexes: several proteins were not found as stable monomer structures on their own *in vivo*, and these proteins are essential for forming complexes [44]. Thus, our interaction data may contribute to further understanding of *P. horikoshii *OT3. Of course, further analysis is necessary to confirm the interactions and the resulting characteristics and predicted functions of the proteins.

## Materials and methods

### Two-hybrid system

Forward and reverse primers specific to the *P. horikoshii *OT3 genes were used to construct the assay samples that expressed the *P. horikoshii *proteins fused with the Gal4 DNA-binding domain (BIND) or the VP16 transcriptional activation domain (ACT). Mammalian two-hybrid assays, including the transfection method, were carried out as previously described [11], with slight modifications. The positive combinations in the assay were categorized by the fold value of luciferase reporter activity as follows: level 1, ≥ 3 to <5 times as high as the background activity; level 2, ≥ 5 to <10 times as high; and level 3, ≥ 10 times as high. For a more detailed description, see Additional data file 1.

### Western blot analysis

Bait sample (10 μl) was transfected to 10^5 ^CHO-K1 cells in six-well culture plates using Lipofectamine2000 (Invitrogen, Carlsbad, CA, USA). After 24 h of incubation, cells were washed once with ice-cold TBS (50 mM Tris-HCl, pH 8.0, 137 mM NaCl, 2.68 mM KCl) and harvested using 200 μl of Lamuli sample buffer. The sample was boiled for five minutes and suspended with vortex mixer for 30 s. Protein in Lamuli sample buffer (10 μl) was subjected to 12% SDS-PAGE and transferred electrically onto a polyvinylidene fluoride (PVDF) membrane. The membrane was blocked by TBS/0.05% w/v Tween 20 (TBS-T) containing 6% w/v skim milk for 1 h and incubated with a polyclonal antibody against the Gal4 DNA binding domain (dilution 1:200; Santa Cruz Biotechnology, Santa Cruz, CA, USA) for 1 h. After washing with TBS-T, the membrane was incubated with horse radish peroxidase (HRP)-conjugated anti-rabbit goat IgG (dilution 1:2,000; GE (Amersham Biosciences, Piscataway, NJ, USA) for 1 h and washed with TBS-T. Detection of the signal was performed using the ECL plus system (GE Amersham Biosciences).

### *In vitro *pull-down assay

*In vitro *pull-down assays were carried out as previously described [45] with slight modifications. The template DNA was constructed using overlapping PCR, which has the T7 promoter sequence upstream of the *P. horikoshii *genes. Biotinylated or ^35^S-labeled proteins were synthesized *in vitro *according to the manufacturer's protocols, using the Transcend Biotinylated lysine-tRNA (Promega, Madison, WI, USA), redivue L-[^35^S] methionine (Amersham Biosciences), and TNT^® ^T7 Quick Coupled Reticulocyte Lysate system (Promega). The samples were applied to the Centrisep spin column (Applied Biosystems, Foster, CA, USA) and diluted with an equal volume of 1 × phosphate-buffered saline without 1 mM CaCl_2 _and 0.5 mM MgCl_2 _(PBS (-)). Each sample was divided into two microcentrifuge tubes and incubated for 15 minutes at 37°C or 75°C (non-heat and heat, respectively). Samples were then centrifuged at 15,000 × *g *for 20 minutes at 4°C and the supernatant collected. Recovery of ^35^S-labeled proteins in the non-heat and heat samples was estimated using SDS-PAGE followed by autoradiography. Equal amounts of biotinylated and ^35^S-labeled proteins were mixed and incubated for 1 h at 25°C. Dynabeads^® ^Streptavidin (Dynal Biotech LLC, Milwaukee, WI, USA) were added to the reaction mix and incubated on a rotary shaker for 30 minutes at 25°C. The beads were isolated with the magnet and washed three times with ice-cold TBS-T. The precipitated proteins were subjected to SDS-PAGE followed by autoradiography.

### Calculation of the interaction generality

The IG was calculated with reference to its definition [20]. The IG value of the PPI between proteins *a *and *b *is defined as:



where *N *is a set of proteins such that *x, y, a, b *∈ *N*. *E *is the set of PPIs between proteins *a *and *b*. In the calculation, we considered the protein *a *(BIND)-protein *b *(ACT) interaction and the protein *b *(BIND)-protein *a *(ACT) interaction to be a single interaction (a bi-directional interaction in a two-hybrid experiment). Furthermore, we removed the self-interactions from the calculation of the IGs. Therefore, *E *is the set with conditions such that ∀*a,b*{(*a,b*) ∈ *E *∧ (*b,a*) ∈ *E*} and ¬∃*x*{(*x,x*) ∈ *E*}.

### Ortholog search and classification

Orthologs for each *P. horikoshii *OT3 protein were identified using the Sequence Similarity Database (SSDB) [46] and the Gene Function Identification Tool (GFIT) program [47] in the Kyoto Encyclopedia of Genes and Genomes (KEGG) [48,49]. We screened the orthologous protein sequences from other organisms by using *P. horikoshii *OT3 proteins as query sequences and selected those that exhibited a Smith-Waterman score of 200 or more.

### Data availability

All PPI data from *P. horikoshii *OT3 have been stored in a database supported by DDBJ [50]. These interaction data have also been deposited with each BIND ID (331136 to 331235) in the Biomolecular Interaction Network Database (BIND) [51], as well as 100 single interaction records (not including reciprocal interaction data), and 3 records determined from *in vitro *pull-down assays (Figure 3; BIND IDs 331124 (PH0245-PH1533), 331125 (PH0795-PH0017) and 331126 (PH1354-PH1355).

## Additional data files

The following additional data are available with the online version of this article. Additional data file 1 includes detailed methods for the PPI mammalian two-hybrid system assay. Additional data file 2 is a spreadsheet including four lists showing the experimental raw data as follows: sheet 1, a list of 1,390 clones of *P. horikoshii *OT3 including results from the SOSUI program; sheet 2, a list of 980 clones that were used for the interaction assay in this study; sheet 3, a list of 170 primary protein interactions with their fold values for luciferase activity; sheet 4, a list of 107 selected protein interactions.

## Supplementary Material

Additional data file 1 Detailed methods for the PPI mammalian two-hybrid system assay. Click here for file

Additional data file 2 Experimental raw data are as follows: sheet 1, a list of 1,390 clones of *P. horikoshii *OT3 including results from the SOSUI program; sheet 2, a list of 980 clones that were used for the interaction assay in this study; sheet 3, a list of 170 primary protein interactions with their fold values for luciferase activity; sheet 4, a list of 107 selected protein interactions. Click here for file

## Figures and Tables

**Table 1:** The selected self-interaction proteins

	Annotated or functional predicted proteins		Hypothetical proteins
	Gene ID	Function	Gene ID

Ortholog to eubacteria, eukaryote and archaea proteins	PH0125*	5'-methylthioadenosine phosphorylase	PH1904
	PH0413*	Glucose-1-phosphate thymidylyltransferase	
	PH0540*	S-adenosyl-L-homocysteine hydrolase	
	PH0655*	Threonine-3-dehydrogenase	
	PH1355	SNZ like protein	
	PH1589*	Deoxycytidylate deaminase	
	PH1884*	Triosephosphate isomerase	
			
Ortholog to eubacteria and archaea proteins	PH0385	MorR-like ATPase	PH0008
	PH0596*	Pyrrolidone-carboxylate peptidase	PH1305
	PH0634*	6-pyruvoyl-tetrahydropterin synthase	PH1503
	PH0762*	Probable thymidylate synthase	PH1950
	PH0776	Methanol dehalogenase regulatory protein	PH0519
	PH0986^†^	Lactam utilization protein	PH1474
	PH1153*	Putative acetylornithine deacetylase	PH1918
	PH1408	Putative uridylate kinase	
	PH1692*	Transcriptional regulator (AsnC-like protein)	
	PH1821*	Frv operon protein, endoglucanase	
	PHS023*	Regulatory protein AsnC	
			
Ortholog to eukaryote and archaea proteins	PH0119*	DNA repair protein (RadA/Rad51)	PH1257
	PHS042*	Small nucleoprotein	
			
Archaea-specific proteins	PHS053*	Archaea-specific DNA-binding protein AlbA	PH0073
			PH0130^†^
			PH0197
			PH0250^†^
			PH0406
			PH0795
			PH1025
			PH1120
			PH1528^†^
			PH1895^†^
			PHS017^†^
			PH0127
			PH0187
			PH0223
			PH0346^†^
			PH0690
			PH0860^†^
			PH1191^†^
			PH1126^†^
			PH1850^†^
			PH1931

The gene identity code (ID) refers to that of Database of Genomes Analyzed in NITE (DOGAN) [26] entries for *P. horikoshii *OT3 [26]. Ortholog classification was performed using the Sequence Similarity Database (SSDB) [46] in the Kyoto Encyclopedia of Genes and Genomes (KEGG) [48,49]. *The proteins were reported to form a homo-oligomer in *P. horikoshii *OT3 or orthologous proteins (interlogs of self-interaction). ^†^Proteins unique to *Pyrococcus *without orthologs in another archaea species.

**Table 2:** Interacting proteins encoded at adjacent loci on the *P. horikoshii *OT3 genome

Protein 1		Protein 2	
ORF	Annotation	ORF	Annotation

PH1354	SNO like protein	PH1355	SNZ like protein
PH0487	Chemotaxis protein CheC	PH0490	Chemotaxis protein CheD
PH1978	AtpE, archaeal or vacuolar-type H^+^-ATPase subunit E	PH1983	AtpH, archaeal or vacuolar-type H^+^-ATPase subunit H
PH0073	Hypothetical protein (Paralog to PH0074)	PH0074	Hypothetical protein (Paralog to PH0073)
PH0812	Hypothetical protein (Paralog to PH0813)	PH0813	Hypothetical protein (Paralog to PH0812)
PH0126	3-isopropylmalate dehydratase	PH0127	Hypothetical protein
PH0353	Hypothetical protein	PH0354	Hypothetical protein
PH0402	Hypothetical protein	PH0403	Hypothetical protein
PH0468	Hypothetical protein	PH0469	Hypothetical protein
PH1025	Hypothetical protein	PH1024	Hypothetical protein
PHS014	Hypothetical protein	PHS013	Hypothetical protein

ORF, open reading frame.
