# Supplementary material for: Protein-protein interactions of the hyperthermophilic archaeon Pyrococcus horikoshii OT3
Source: Genome Biol. 2005 Nov 18;6(12):R98. doi: 10.1186/gb-2005-6-12-r98 (PMC1414084; doi:10.1186/gb-2005-6-12-r98)
Supplement: Additional data file 1 — Detailed methods for the PPI mammalian two-hybrid system assay. [file gb-2005-6-12-r98-S1.pdf]

Additional file 1

Protein-protein interaction assay by mammalian 2-hybrid system.

Primers

The forward primers specific to each ORF were designed to have a consensus tag sequence, 5’-AGCGAGAAGGAGATATACAT-3’, followed by a 20-base 5’-end ORF sense-stranded sequence. Each reverse primer was designed to have another tag sequence, 5’-AAAGAGGTAATGAAATGGCA-3’, followed by a 20-base 3’-end ORF anti-sense-stranded sequence. Other primers used in this work were as follows: RSALSE-PH, 5’-**ATGTATATCTCCTTCTCGCT**TCGCGTCAAGTCGACT-3’; FSV40LPAS02-PH, 5’-**TGCCATTTCATTACCTCTTT**ATCCGCTGCAGACATGATAAGATACATTG-3’; FPCMV6, 5’-CCAATATGACCGCCATGTTGGC-3’; its nested primer FPCMV5, 5’-GCCATGTTGGCATTGATTATTGAC-3’; and RSV40LPAS01, 5’-AGCAAGTTCAGCCTGGTTAAGATCCTTATCGATTTTACCAC-3’. The sequences in bold type in RSALSE-PH and FSV40LPAS02-PH are complementary to the common tag sequences of the ORF-specific forward and reverse primers, respectively.

Construction of the assay samples

Plasmid vectors pACT and pBIND were from the CheckMate mammalian two-hybrid system (Promega Co.). Fragments for the CMV promoter and the Gal4 DNA-binding domain (BIND) or the VP16 transcriptional activation domain (ACT) were PCR-amplified from pBIND or pACT vectors using the primer sets FPCMV6 and RSALSE-PH. Fragments for the SV40 late polyadenylation signal (SV40LPAS) were PCR-amplified from pBIND vectors using the primer sets FSV40LPAS02-PH and RSV40LPAS01. First, each ORF of *P. horikoshii* OT3 was amplified using the corresponding ORF-specific forward and reverse primers. Overlapping PCR was then carried out to obtain the assay constructs, in which each ORF fragment was connected with the BIND or ACT fragments at the 5'-end of the first PCR product and the SV40LPAS fragment at the 3'-end. One microliter of each of the ORF fragments was mixed with 0.5 μl of BIND or ACT fragments and SV40LPAS fragments, and amplified in 100-μl reactions using the primer sets FPCMV5 and RSV40LPAS01. All the PCR conditions were based on those in our previous report [1].

Two-hybrid assay

Mammalian two-hybrid assays, including the transfection method, were carried out as previously described [1], with slight modifications. Briefly, the assay was separated into three phases—the BIND pre-assay, the first assay, and the final assay. In the BIND (bait) pre-assay, in order to remove BIND samples with high self-activation, each BIND sample was transfected to CHO-K1 cells together with the luciferase reporter plasmid pG5*luc*, and

the reporter activity was measured after 20 h incubation. In the first assay, two-mixture of BIND and two-mixture of ACT (prey) samples together with pG5*luc* were transfected to the cells, and then the luciferase activity was measured. We selected sample combinations with luciferase activity significantly higher than the background activity. In the final assay, positive combinations of single BIND samples and single ACT samples were determined. Finally, the positive combinations in the final assay were categorized by the fold value of luciferase activity as follows: level 1, ≥3 to <5 times as high as the background activity; level 2, ≥5 to <10 times as high; and level 3, ≥10 times as high.

Reference

1. Suzuki H, Fukunishi Y, Kagawa I, Saito R, Oda H, Endo T, Kondo S, Bono H, Okazaki Y, Hayashizaki Y: **Protein-protein interaction panel using mouse full-length cDNAs**. *Genome Res* 2001, **11:**1758-1765.
